# Recent Developments in Studies on the Relation Between Endometriosis and Ovarian Cancer: Phenotype-Specific Risk, Molecular Convergence and Clinical Risk Stratification

**DOI:** 10.3390/cancers18193241

**Published:** 2026-10-08

**Authors:** Klaudia Gutowska, Aleksandra Urban, Cezary Wojtyła, Sławomir Ławicki, Piotr Laudański

**Affiliations:** 1 Department of Obstetrics and Gynecology, Institute of Mother and Child, 01-211 Warsaw, Poland; 2 2nd Department of Obstetrics and Gynecology, Medical University of Warsaw, 02-091 Warsaw, Poland; 3 Women’s Health Research Institute, Calisia University, 62-800 Kalisz, Poland; 4 OVIklinika Infertility Center, 01-377 Warsaw, Poland; 5 Department of Population Medicine and Lifestyle Diseases Prevention, University of Białystok, 15-269 Białystok, Poland; 6 Department of Obstetrics, Gynecology and Gynecological Oncology, Medical University of Warsaw, 03-242 Warsaw, Poland

**Keywords:** endometriosis, endometrioma, ovarian cancer, clear-cell carcinoma, endometrioid carcinoma

## Abstract

**Background/Objectives**: We aim to synthesize recent clinical and translational evidence on the relationship between endometriosis and ovarian cancer, with emphasis on publications from 2024 to 2026 and on clinically conservative risk stratification. **Materials and Methods**: This state-of-the-art narrative review prioritizes major peer-reviewed publications from July 2024 to June 2026. Older landmark publications were used only where necessary to contextualize recent findings. **Results**: Recent evidence supports a selective, phenotype-dependent association instead of a generalized malignant potential of endometriosis. The strongest and most reproducible signal concerns epithelial ovarian cancer, especially clear-cell and endometrioid ovarian carcinoma, with the highest relative risks reported for ovarian endometrioma and/or deep endometriosis. The ENDOCANCER meta-analysis found increased ovarian cancer risk across odds ratio, hazard ratio and standardized incidence ratio estimates but confirmed low absolute incidence and substantial heterogeneity. Contemporary reviews, including Leone Roberti Maggiore et al. and Bogani et al., emphasize low absolute lifetime risk and the absence of evidence supporting universal oncologic surveillance. Newer clinicopathological studies distinguish endometriosis-correlated tumors supported by transitional lesions indicative of malignant transformation from incidental coexistence. Molecular data support biological plausibility through *ARID1A*, *PIK3CA*, *KRAS*, *PTEN*, mismatch repair deficiency, *POLE*-mutated disease and p53-abnormal phenotypes, but these markers are not ready for routine prediction in endometriosis care. **Conclusions**: The immediate clinical implication is individualized risk assessment in place of routine cancer screening or prophylactic surgery based solely on the increased cancer risk associated with endometriosis. Counseling should integrate endometriosis phenotype, age, menopausal status, lesion dynamics, imaging atypia, hereditary cancer risk, reproductive goals and surgical consequences.

## Simple Summary

Endometriosis is a chronic disorder in which tissue similar to the uterine lining grows outside the uterus, often causing pain and fertility problems. Researchers have long asked whether it also raises ovarian cancer risk. This review summarizes the most important studies from the last two years to answer that question. Oncology-related risk is elevated mainly in certain forms of the disease, particularly ovarian cysts caused by endometriosis and deeper growths. Importantly, the overall chance of developing cancer remains low. Current evidence supports a more personalized approach that considers a woman’s age, menopausal status, family history and changes in the disease over time.

## 1. Introduction

Endometriosis is a chronic estrogen-dependent inflammatory disease defined by endometrial-like tissue outside the uterine cavity. Although histologically benign, it can show cancer-like properties, including local invasion, resistance to apoptosis, neoangiogenesis, immune modulation and recurrence after incomplete treatment. These features have long encouraged comparison with neoplasia, but the clinically relevant question is more specific: which cancers are reproducibly associated with endometriosis, in which phenotypes, and with what absolute risk?

This review focuses on studies published over the past two years, a period marked by a growing number of reports examining the association between endometriosis and cancer. As the available evidence remains inconsistent, an up-to-date synthesis of these findings is warranted. More specifically, evidence indicates that risk is concentrated in a restrictive model in which risk is concentrated in epithelial ovarian cancer, particularly type I tumors and especially clear-cell ovarian carcinoma and endometrioid ovarian carcinoma. On the other hand, reported associations with breast, endometrial, thyroid or melanoma risk remain more vulnerable to detection bias, reproductive confounding, hormonal treatment patterns, infertility, adenomyosis and health-care utilization.

This article summarizes the major recent publications that should inform a clinically oriented state-of-the-art synthesis. The central argument is that endometriosis-related cancer risk should be conceptualized through phenotype-specific and histotype-specific risk stratification, not through universal oncologic surveillance. The 2026 review by Leone Roberti Maggiore et al. provides the core clinical synthesis, concluding that ovarian endometrioma is associated with an approximately two-fold increased ovarian cancer risk, particularly for clear-cell and endometrioid subtypes, while the absolute lifetime risk remains relatively low, estimated at about 1.9% [1].

## 2. Materials and Methods

This is a focused narrative review. The two-year scope was chosen because this period opened with the first large population-based cohort to report ovarian cancer risk separately by endometriosis phenotype, followed by a meta-analysis, multidisciplinary reviews and clinicopathological studies that changed how this risk is quantified and interpreted [1,2,3,4]. The priority interval was 1 July 2024 to 30 June 2026, defined by the date of first online publication. Earlier studies were retained when needed to interpret recent findings. Corrections to included articles were checked irrespective of publication date. Articles published after this interval (July–September 2026) were added during revision only when they directly addressed questions raised in peer review.

Keywords included combinations of “endometriosis”, “endometrioma”, “deep endometriosis”, “ovarian cancer”, “clear cell”, “endometrioid”, “ARID1A”, “PIK3CA”, “single-cell”, “multi-omics”, “assisted reproductive technology” and “risk stratification”.

Searches were conducted in major databases, including PubMed, Embase and Wiley Online Library. English-language, peer-reviewed reports addressing ovarian cancer risk, histotype or phenotype differences, malignant transformation, or clinically relevant biomarkers were selected. Titles and abstracts were assessed for relevance, followed by examination of relevant article sections where accessible. Isolated case reports and unreviewed preprints were not included in the review. Multi-omics studies were used only for methodological context. Studies were selected based on their relevance to the aims of this review, and the search was not intended to be exhaustive. No duplicate screening nor formal risk-of-bias assessment was carried out.

The review is deliberately centered on ovarian cancer. This reflects the current evidence hierarchy: the endometriosis–ovarian cancer association is the most consistent, biologically plausible and clinically actionable, whereas many nonovarian associations remain less specific and more prone to bias. The full-text publications by Piacenti et al. and Bogani et al. were used to refine risk estimates, survival interpretation, methodological limitations and clinical management statements [2,3]. The key recent publications are summarized in Table 1.

## 3. Results

### 3.1. Epidemiological Update

The most influential recent epidemiological study is the JAMA population-based cohort by Barnard et al. [4]. Using the Utah Population Database, the authors matched 78,893 women with endometriosis to women without endometriosis in a 1:5 ratio and identified 597 ovarian cancers during follow-up. Endometriosis overall was associated with increased ovarian cancer risk (adjusted hazard ratio [aHR] 4.20, 95% confidence interval [CI] 3.59–4.91). The association was stronger for type I ovarian cancer (aHR 7.48, 95% CI 5.80–9.65) than for type II disease. The highest risk was observed among women with ovarian endometriomas and/or deep endometriosis: aHR 9.66 for all ovarian cancers and aHR 18.96 for type I ovarian cancer [4].

The numerical magnitude of relative risk in Barnard et al. should be interpreted cautiously in counseling. To illustrate it in absolute terms, the adjusted risk difference for ovarian cancer overall was 9.90 additional cases per 10,000 women (95% CI, 7.22–12.57) over a mean follow-up of approximately 12 years. In absolute terms, ovarian cancer occurred in 225 of 78,476 women with endometriosis (approximately 0.29%) compared with 372 of 372,430 women without endometriosis (approximately 0.10%). These figures represent crude cumulative proportions observed during the study follow-up and should not be interpreted as lifetime risk estimates. Large relative increases can coexist with small absolute increases because the baseline incidence of ovarian cancer is low. Correspondence after the article emphasized potential limitations, including dependence on diagnostic coding, incomplete information on hormonal therapy and hereditary cancer risk, and possible detection bias around the time of endometriosis diagnosis [16,17,18]. The authors’ reply reported sensitivity analyses excluding ovarian cancers diagnosed near the time of endometriosis diagnosis, with attenuated but persistent associations [18]. Thus, Barnard et al. should be interpreted as evidence of an increased ovarian cancer risk compared with the general population; however, given the low absolute incidence of the disease and the absence of an established screening strategy for ovarian cancer in the general population, these findings do not support population-based screening in women with endometriosis.

The ENDOCANCER systematic review and meta-analysis by Piacenti et al. provides the most useful recent quantitative synthesis of risk, prevalence and survival [2]. It included 67 observational studies, with risk analyses covering more than 500,000 women and survival analyses covering more than 10,000 patients. Endometriosis was associated with increased ovarian cancer risk across different effect measures: odds ratio 1.82 (95% CI 1.33–2.50), hazard ratio 3.03 (95% CI 2.16–4.25) and standardized incidence ratio 1.62 (95% CI 1.03–2.22). Importantly, absolute incidence remained low at approximately 0.7–1.5%, and heterogeneity was substantial across all pooled risk estimates [2].

ENDOCANCER also provides data on survival narrative. Compared with non-endometriosis-associated ovarian cancer, EAOC (endometriosis-associated ovarian cancer) was more often diagnosed in younger and premenopausal patients, with earlier-stage and lower-grade tumors. Advanced FIGO stage was less frequent (pooled odds ratio 0.29, 95% CI 0.23–0.37), and overall survival appeared better (hazard ratio 0.48, 95% CI 0.32–0.74). However, progression-free survival did not reach statistical significance, and the authors noted moderate risk of bias, temporal ambiguity between endometriosis and cancer diagnosis, heterogeneity in exposure ascertainment and lack of detailed phenotype-specific subgrouping [2]. The survival advantage should therefore be interpreted as probably influenced by stage, histotype, detection patterns and treatment factors.

The approach focused on more frequent imaging and surgical procedures in women with endometriosis is associated with a higher probability of detecting ovarian tumors and can shift diagnosis toward an earlier stage. As a result, this may lengthen measured survival through lead-time bias without reducing mortality. Conversely, endometriosis may first be diagnosed during a cancer screening. In these patients, assessments of survival outcomes need adjustment for age, stage, histotype and treatment [2,4,7,16,17,18].

Bogani et al. [3] reached a concordant clinical conclusion in a multidisciplinary review based on a PRISMA-guided synthesis of 35 risk studies and expert panel discussion. Cohort studies generally reported relative risks between 1.3 and 4.2, with the highest estimates in ovarian endometriomas or deep disease. However, the absolute lifetime risk remained low, generally below 2–5%, and the authors concluded that routine oncologic surveillance or prophylactic surgery is not justified solely by a diagnosis of endometriosis [3].

A Swedish nationwide cohort by Moberg et al. broadened the epidemiological perspective by including women diagnosed with endometriosis or adenomyosis between 1997 and 2018 [5]. Among 49,133 women, the overall standardized incidence ratio for gynecological and breast cancers was 1.4. For subsequent cancers after the diagnostic year, SIRs were 1.9 for epithelial ovarian cancer and 1.2 for breast cancer; ovarian subtype analyses showed stronger associations for clear-cell ovarian cancer (SIR 7.7) and endometrioid ovarian cancer (SIR 2.8) [5]. The combined exposure definition and high same-year cancer rates support cautious interpretation, but the study reinforces histotype specificity.

When interpreting cancer risk, the patient’s age and menopausal status should also be taken into consideration. Moreover, infertility, parity, hormonal treatment and assisted reproductive technology (ART) may confound comparisons. In the Dutch OMEGA cohort, invasive ovarian cancer risk after ART was higher than in the general population but not higher than in subfertile women without ART after adjustment for age and parity (HR 1.02, 95% CI 0.70–1.50) [19]. This finding does not establish the absence of treatment-related risk in every endometriosis subgroup but illustrates why the comparator population matters. Future studies should record menopausal status and the timing of fertility treatment and not attribute the combined effects of infertility and treatment to endometriosis alone [4,19].

### 3.2. Histotype-Specific Risk: Clear-Cell and Endometrioid Ovarian Carcinoma

The contemporary literature consistently identifies clear-cell ovarian carcinoma and endometrioid ovarian carcinoma as the principal histotypes linked to endometriosis. This pattern was visible in older meta-analyses, including the review by Kvaskoff et al., which reported elevated summary relative risks for clear-cell, endometrioid and low-grade serous ovarian cancer but not for high-grade serous or mucinous tumors [20]. Recent studies narrow this further by showing that excess risk is concentrated in ovarian endometrioma and possibly severe/deep phenotypes [1,2,3,4,5].

The ENDOCANCER meta-analysis quantifies this distribution at the tumor level: clear-cell histology was more frequent in EAOC than non-EAOC (29.5% vs. 11.8%), as was endometrioid histology (35.7% vs. 18.9%). Conversely, serous tumors were less frequent in EAOC (24.5% vs. 54.9%), as were mucinous tumors (8.3% vs. 13.2%) [2]. These data are consistent with a type I ovarian cancer framework, but the framework should not obscure biological heterogeneity. Endometrioid and clear-cell carcinomas differ in molecular architecture, treatment response and prognosis.

The biological plausibility is strongest for clear-cell and endometrioid tumors because these histotypes are often identified adjacent to endometriosis or atypical endometriosis and because shared molecular alterations can be detected across benign, atypical and malignant components. However, association must not be confused with inevitability. Most endometriomas do not progress to cancer, and most women with endometriosis will never develop ovarian cancer. Careful diagnostic evaluation is warranted when an ovarian endometrioma presents atypical features, particularly after menopause or when new solid, papillary or vascularized components appear.

An ovarian endometrioma creates a confined cystic space with recurrent bleeding, heme and iron accumulation, oxidative DNA injury and chronic inflammatory mediator exposure. This environment is biologically different from superficial peritoneal implants and may explain the stronger association between endometrioma and clear-cell carcinoma. Deep endometriosis (DE) may mark a severe inflammatory phenotype, but its independent contribution to ovarian carcinogenesis remains difficult to isolate because severe phenotypes often overlap. In the study by Anglesio et al. [21], deep endometriotic lesions from 27 patients were analyzed. Despite the benign nature of the lesions, 10 of 39 deep endometriotic lesions were shown to harbor somatic driver mutations in genes such as KRAS, PIK3CA, ARID1A, and PPP2R1A, with these alterations confined to the epithelial rather than the stromal component. Nevertheless, no reliable molecular predictors or well-established clinical indicators have yet been identified to determine which women with endometriosis are at the highest risk of malignant transformation [22].

Direct comparison of the three principal phenotypes shows both a consistent ovarian signal and important uncertainty outside the ovary. In the Finnish surgical cohort, ovarian endometriosis was associated with clear-cell carcinoma (SIR 10.1, 95% CI 5.50–16.9) and endometrioid carcinoma (SIR 4.72, 95% CI 2.75–7.56). Peritoneal disease showed no statistically significant increase in ovarian cancer overall, although endometrioid carcinoma was increased (SIR 2.03, 95% CI 1.05–3.54). DE was not associated with a statistically significant excess, but only three ovarian cancers occurred in that subgroup [23]. By contrast, the Utah cohort reported elevated risk in DE, although precision was limited [4].

### 3.3. Endometriosis-Correlated Versus Incidental Ovarian Cancer

A major conceptual advance in 2025–2026 is the separation of endometriosis-associated ovarian cancer into correlated and incidental entities. Mezzapesa et al. [6] classified ovarian cancers into endometriosis-correlated tumors, endometriosis-incidental tumors and ovarian cancers without endometriosis. Correlated tumors were defined by transitional elements, including atypical endometriosis or borderline tumors, implying a plausible lesion-to-carcinoma sequence. In a cohort of 170 patients, correlated tumors were more likely to be early-stage, unilateral, multilocular-solid and of clear-cell, endometrioid or mesonephric-like histology [6].

Beck et al. applied a related framework in a real-world cohort from an ESGO-accredited center [7]. Among 2164 patients with malignant or borderline ovarian tumors, 176 had histologically confirmed endometriosis, including 22 endometriosis-correlated and 154 endometriosis-incidental ovarian tumors. Patients with endometriosis were younger and more often had early-stage disease and endometrioid or clear-cell histology. Correlated tumors showed a high prevalence of ovarian endometriosis, absence of p53 aberrations and no BRCA1/2 mutations. After multivariable adjustment, correlated or incidental status was not independently associated with overall or disease-free survival; prognosis was driven mainly by age, FIGO stage, tumor biology and completeness of cytoreduction [7].

This distinction helps avoid attributing every cancer to endometriosis. Not every ovarian cancer found in a patient with endometriosis arises from endometriosis. It also explains conflicting survival studies: apparent prognostic advantages in EAOC may be confounded by earlier stage and overrepresentation of non-serous histotypes. Future studies should report correlated and incidental disease separately whenever tissue architecture allows.

Comparisons with high-grade serous ovarian carcinoma (HGSOC) lead to a similar conclusion about prognosis. In a 10-year single-center cohort of 224 patients from Taiwan, Sung et al. compared 102 clear-cell or endometrioid carcinomas, classified as EAOC, with 122 HGSOC cases [8]. Patients with EAOC were younger (mean age 49.2 vs. 56.5 years) and more often had early-stage disease (70.6% of EAOC cases, while 84.4% of HGSOC cases were stage III-IV). They also more often achieved optimal cytoreduction (84.3% vs. 50.8%), and their crude overall and progression-free survival was better. However, the survival advantage diminished after stratification by stage and platinum sensitivity. No patient with platinum-resistant EAOC survived beyond 5 years, but a subset of patients with platinum-resistant HGSOC achieved long-term survival [8]. Histologically confirmed endometriosis was present in only 45.1% of the EAOC group, and coexisting endometriosis was not an independent prognostic factor in multivariable analysis. As EAOC was defined by histological type and not by confirmed endometriosis, the study could not distinguish tumors arising from endometriosis from those that only coexisted with it. Nonetheless, it supports the view that the favorable crude prognosis of EAOC largely reflects stage and surgical outcome and that chemoresistance, particularly in clear-cell carcinoma, remains a key determinant of poor survival [7,8].

### 3.4. Molecular Mechanisms

Recent molecular publications support a multi-step, context-dependent molecular model in the context of the malignant process. Recurrent alterations include *ARID1A* loss, *PIK3CA* and *KRAS* mutations, PI3K/AKT/mTOR pathway activation, *PTEN* dysfunction, mismatch repair deficiency, *POLE*-mutated tumors and p53-abnormal phenotypes [1,3,17,18,19]. These changes are not uniformly distributed across histotypes. They are most relevant to clear-cell and endometrioid tumors, but p53-abnormal biology is more typical of aggressive high-grade serous carcinoma and may indicate a different oncogenic route.

*ARID1A* is the best-established molecular link between endometriosis and EAOC. It encodes a component of the SWI/SNF chromatin remodeling complex, and loss-of-function alterations have been observed in clear-cell and endometrioid carcinomas as well as in adjacent atypical endometriosis. Bogani et al. summarize that *ARID1A* mutations occur in approximately 50% of ovarian clear-cell carcinomas and 30% of endometrioid ovarian carcinomas, supporting early involvement in a subset of tumors [3]. Nevertheless, *ARID1A* loss alone is insufficient for malignant transformation; the endometrioma microenvironment probably adds selective pressure through hemorrhage, iron overload, oxidative stress, inflammation and altered immune surveillance [1,3].

Two further publications offer a new perspective on this problem. In the first, Santhamma et al. [10] presented a multi-mechanism model of disease initiation and progression. In this model, endometriosis is driven by the clonal expansion of cells carrying somatic driver mutations in ARID1A, PIK3CA, KRAS and PTEN, together with dysregulated PI3K/AKT/mTOR signaling. Estrogen dominance combined with progesterone resistance, as well as complex pro-inflammatory and fibrotic networks in the lesion microenvironment, also play an important role in this process. In the second publication, Umezaki et al. proposed a coupled iron–hypoxia selection model of clear-cell carcinoma development [11]. According to this paper, chronic intracystic hemorrhage and low oxygen tension act as a ferroptosis-related selection filter within the endometrioma. This selective pressure is hypothesized to favor precursor epithelial cells that develop robust antioxidant defenses mediated by the NRF2/SLC7A11/GPX4 axis. It may also favor cells that tightly regulate their labile iron pool and undergo glycolytic reprogramming driven by HIF-1α/GLUT1 and HNF-1β. In addition, the surviving cells may activate compensatory survival pathways after loss of ARID1A. These studies indicate that malignant transformation results from the combined action of somatic driver alterations, persistent oxidative stress and immune–stromal remodeling. Importantly, cancer-associated mutations may already be present in histologically benign epithelium [21]. Studies of clear-cell carcinoma and endometriosis showed that PIK3CA mutations and loss of ARID1A protein had occurred before cancer invasion [24]. However, neither alteration is restricted to atypical endometriosis, and no fixed sequence of molecular alterations has been established. PIK3CA activation and loss of PTEN-mediated inhibition concentrate on the PI3K/AKT/mTOR pathway, while KRAS provides a parallel proliferative path. In mice, inactivation of ARID1A alone did not lead to tumor formation. However, when ARID1A inactivation was combined with PIK3CA activation, the animals developed tumors resembling clear-cell carcinoma, accompanied by persistent IL-6 activation [25].

Ioannidou et al. [26] emphasized that *ARID1A* alterations, microsatellite instability, PI3K/AKT/mTOR dysregulation, oxidative stress, mismatch repair deficiency and epigenetic changes recur across the malignant transformation literature. Ye et al. extended this discussion by applying TCGA-style molecular classification to EAOC: endometrioid ovarian carcinoma associated with endometriosis had higher frequencies of *POLE*-mutated and mismatch repair-deficient subtypes than clear-cell carcinoma, while p53-abnormal status was associated with worse disease-free or progression-free outcomes [9].

Experimental evidence supports the need for multiple cooperating hits. Sohel et al. developed in vitro and in vivo models of endometriosis-related ovarian neoplasms using engineered human endometrial cells. Combinations involving *ARID1A* knockout, *KRAS* activation, AKT overexpression and *MYC* overexpression generated malignant tumors in immunosuppressed mice, and tumor morphology varied with the immune context [27]. This supports a multi-step model: susceptible lesion phenotype, local inflammatory and oxidative microenvironment, acquisition of permissive molecular alterations and, in a minority of cases, emergence of atypical endometriosis before carcinoma.

Linder et al. performed whole-exome sequencing of ovarian endometriosis and subsequently diagnosed ovarian carcinoma samples with a median interval of approximately 10 years between endometriosis surgery and cancer diagnosis. The degree of clonal dominance (variant allele frequency) in the precursor lesion correlated with time to malignant transformation, suggesting that clonal evolution within endometriotic lesions is a temporally extended process, not a sudden event [28]. Separately, increased expression of the mTOR-related genes AKT1 and 4EBP1 was detected in women with early-stage endometriosis [29], while proangiogenic AKT1 upregulation was observed in women with advanced ovarian endometriosis [30]. However, these studies examined only eutopic endometrium.

**Figure 1:**
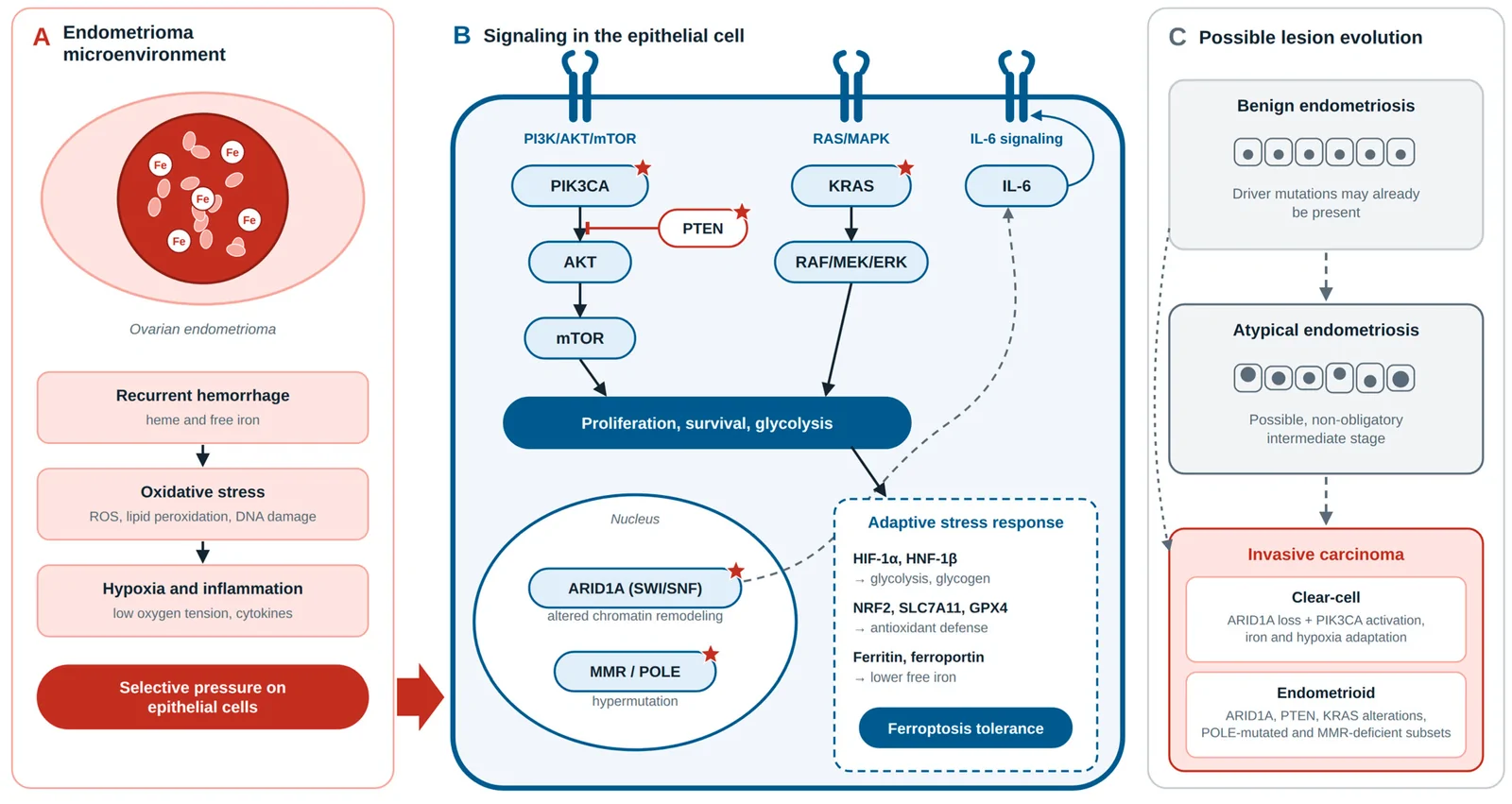
Molecular pathways implicated in endometriosis-associated ovarian carcinogenesis. (**A**) In an ovarian endometrioma, recurrent hemorrhage releases heme and free iron. Iron catalyzes the formation of reactive oxygen species (ROS), which leads to lipid peroxidation. Together with hypoxia and chronic inflammation, these processes exert selective pressure on the epithelium. (**B**) In epithelial cells, activating PIK3CA mutations and loss of PTEN function converge on the PI3K/AKT/mTOR pathway, and KRAS mutations provide parallel RAS/MAPK signaling. Loss of ARID1A, a component of the SWI/SNF complex, alters chromatin remodeling. In a mouse model, combined ARID1A loss and PIK3CA activation sustained IL-6 signaling. Mismatch repair (MMR) deficiency and POLE mutations define hypermutated tumor subsets. Adaptive programs involving HIF-1α, HNF-1β, NRF2, SLC7A11, GPX4 and iron-handling proteins may allow selected cells to tolerate ferroptosis-related stress. This adaptive stress response should be considered as a model proposed by the authors. (**C**) Possible sequence from benign endometriosis to atypical endometriosis and invasive clear-cell or endometrioid carcinoma. Solid black arrows indicate activation or signal transmission, and the red line with a blunt end indicates inhibition. The red block arrow between panels (**A**) and (**B**) represents the selective pressure exerted by the lesion microenvironment on epithelial cells. Red stars indicate genes with recurrent somatic alterations. Dashed arrows point out links supported mainly by experimental models, such as the effect of combined ARID1A and PIK3CA alterations on IL-6 signaling, as well as possible but non-obligatory steps of lesion progression. The curved dashed arrow in panel (**C**) indicates that carcinoma may also develop without a recognizable atypical stage. The dashed frame marks the hypothetical adaptive stress response.

Multi-omics approaches can integrate genomic findings with other molecular layers and thus provide a more complete picture of this process. Yang et al. reanalyzed single-cell RNA sequencing data from 14 donors (21,600 cells), together with bulk transcriptomes from 24 biopsies of control endometrium, eutopic endometrium, and peritoneal and ovarian lesions [12]. Inflammatory and metabolic programs varied by tissue and cellular compartment. Compared with control endometrium, 4632 genes were differentially expressed in ovarian lesions and 4480 in peritoneal lesions; however, only 31 genes differed in eutopic endometrium. In myeloid cells, inflammatory activity correlated with glycolysis (rho = 0.52), but the association in bulk biopsies was not significant (rho = 0.21) [12]. The authors also independently analyzed endometrial carcinoma data from The Cancer Genome Atlas (TCGA-UCEC). These data did not support an endometriosis-to-cancer progression mechanism [12]. Using an integrative bioinformatic analysis of public transcriptomic datasets, Dou and Xia identified SLC12A8 as a gene upregulated in both endometriosis and EAOC, and they validated its function in vitro [13]. In patients with EAOC, higher SLC12A8 expression was associated with a poorer prognosis. In cell line experiments, silencing or overexpressing SLC12A8 changed the proliferation, migration and invasion of both endometriosis and EAOC cells. Together, these studies suggest that informative signals may be confined to specific cell compartments and diluted in bulk tissue. The next step would be an integrated, multi-omics approach to understanding how the inflammatory and oxidative microenvironment leads to clonal expansion and malignant transformation. Such an approach should combine genomic profiling of ARID1A, PIK3CA, KRAS and PTEN with single-cell transcriptomics, DNA methylation, proteomics, metabolomics and spatial analyses; strategies of this kind are increasingly used in biomedical research [31]. In addition, benign, atypical and malignant components should be sampled from the same patients and followed longitudinally. The discussed pathways and the proposed lesion evolution are summarized in Figure 1.

### 3.5. Biomarkers and Clinical Management

The studies published between 2024 and 2026 may encourage broader surveillance. Current evidence does not justify that step for all women with endometriosis. CA-125 is frequently elevated in endometriosis and lacks specificity; HE4 and ROMA may improve discrimination in women with an adnexal mass, but they are not validated as population screening tools for women with endometriosis. Bogani et al. cite sensitivities of 89–93% and specificities of 87–91% for CA-125 plus HE4/ROMA in women with pelvic masses yet also state that no evidence supports routine oncologic surveillance or prophylactic surgery solely because of endometriosis [3].

Although a previous study identified significant changes in selected miRNAs in women with advanced ovarian endometriosis [32], providing insight into altered endometrial function, microRNAs are not yet ready for clinical implementation. The 2026 systematic review by Szubert et al. screened 2387 manuscripts and included 13 studies comprising 608 patients and more than 1000 miRNAs [14]. No consistent miRNA up- or downregulation pattern was observed across studies, and meta-analysis was not feasible because of heterogeneity and limited raw data.

Available evidence is insufficient to establish a miRNA set capable of distinguishing endometriosis from EAOC or predicting carcinogenesis [14].

Circulating protein complexes involved in the pathogenesis of cancer and autoimmune diseases have also been investigated as potential biomarkers. Using surface plasmon resonance, levels of the 20S proteasome and the 20S immunoproteasome were measured in women with and without endometriosis. The analysis showed lower plasma proteasome levels in early-stage disease and higher plasma immunoproteasome levels in women with ovarian cysts but did not establish clinical utility as a biomarker. These findings warrant further investigation of their biological role in endometriotic lesions [33].

Systemic markers of biological aging have also been examined. Kordowitzki et al. compared blood-based DNA methylation age, telomere length and age-related CpG methylation in 49 women with endometriosis, 11 patients with EAOC and 39 unaffected controls [15]. Women with endometriosis showed epigenetic age acceleration (+16.92%) with relatively long telomeres (13.18 ± 0.46 kbp); however, patients with EAOC appeared biologically younger than their chronological age (−14.64%) but had shorter telomeres (12.45 ± 0.72 kbp) [15]. The authors interpreted these findings as distinct aging trajectories in the two conditions. However, the EAOC group was small and considerably older than the endometriosis cohort, so direct comparisons were limited to an age-matched subset of 24 women aged 35 years or older; the design was retrospective, and blood from patients with EAOC was collected at the time of primary treatment. Therefore, these findings show differences between patients who already have endometriosis or EAOC. They cannot be used to predict whether malignant transformation will occur in an individual patient.

**Figure 2:**
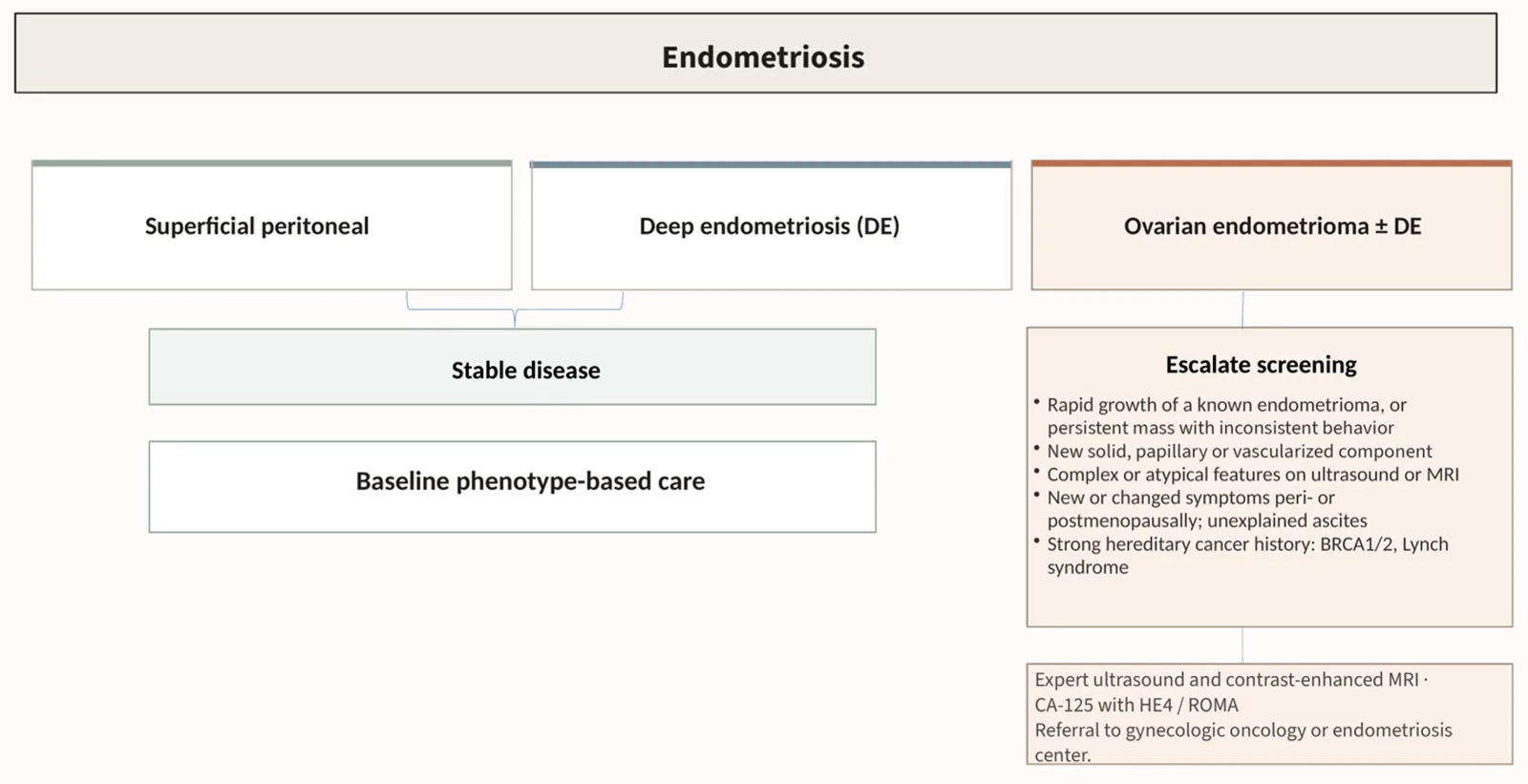
Proposed clinical algorithm for ovarian cancer risk stratification in endometriosis. Stable superficial peritoneal and deep disease should follow baseline phenotype-based care. Stable ovarian endometriomas without suspicious features also require individualized endometriosis care, not routine cancer screening. Ovarian endometrioma ± DE warrants diagnostic reassessment when the highlighted clinical or imaging features are present. Assessment begins with expert ultrasound, with MRI for indeterminate findings and selective use of CA-125 or HE4/ROMA in the evaluation of an adnexal mass. Referral to gynecologic oncology or an endometriosis center depends on the imaging findings and clinical context [1,2,3,34].

A practical approach is to separate baseline counseling from diagnostic escalation (Figure 2). Baseline counseling should state that the risk signal is real but uncommon, strongest for endometrioma and selected ovarian histotypes, and not equivalent to a diagnosis of premalignancy. Diagnostic escalation should be triggered by clinical change: rapid growth of a known endometrioma, complex or atypical ultrasound features, new symptoms in peri- or postmenopausal women, persistent masses with inconsistent behavior, atypical MRI features, unexplained ascites, or strong hereditary cancer history [2,3].

Management should therefore be individualized. Relevant modifiers include endometriosis phenotype, age and menopausal status, persistence or growth of an adnexal lesion, new solid or vascular components, infertility history, previous ovarian surgery, family history, BRCA1/2 or Lynch syndrome risk, planned hormonal therapy, fertility goals and surgical consequences. Long-term combined oral contraceptive use may reduce ovarian cancer risk and can be considered when otherwise clinically appropriate but should not be framed solely as cancer prophylaxis [1,3]. Surgery for endometrioma must balance possible risk reduction against ovarian reserve loss, adhesions and recurrence. Prophylactic salpingo-oophorectomy is appropriate for established hereditary cancer predisposition in guideline-based contexts but is not routinely indicated for endometriosis alone [1,2,3].

For clinical use, risk stratification should begin with information obtainable at the visit: lesion site and phenotype, age, menopausal status, family history, prior pathology, and standardized ultrasound findings compared with earlier examinations. Histological atypia, when tissue is available, warrants expert pathological review and individualized multidisciplinary assessment; tissue sampling solely to obtain a molecular risk profile is not established practice. Stable lesions without concerning features should receive individualized endometriosis care, while a new vascularized solid component, suspicious interval change or an indeterminate mass should prompt expert adnexal assessment, with MRI when needed [1,2,3,34]. This is diagnostic evaluation of a lesion, not population cancer screening. Models incorporating molecular features should distinguish the probability of malignancy already present from future transformation risk and demonstrate calibration, external validity and clinical benefit before they determine follow-up intervals or surgery.

Sorrentino et al. propose a molecularly guided clinical work-up in which somatic driver mutations identified in endometriotic tissue or cyst-fluid liquid biopsy (e.g., ARID1A loss, PIK3CA or PTEN alterations) are used to escalate surveillance intensity, shifting selected patients from routine ultrasound follow-up to contrast-enhanced MRI and closer monitoring, with surgical management considered when mutations suggest oncogenic potential [22]. For now, this approach remains conceptual, but it illustrates the direction in which risk stratification is likely to evolve. No mutation-based threshold for surveillance or surgery has been prospectively validated.

The strongest justification is not the diagnosis of endometriosis itself but a changing or atypical adnexal lesion in a patient whose clinical context increases pre-test probability. In that scenario, referral to an experienced gynecologic oncology or endometriosis center is more rational than serial nonspecific testing in stable low-risk disease.

## 4. Conclusions

The last two years have led to a more clinically useful model. Endometriosis is associated with ovarian cancer, but the association is more selective than global. The strongest evidence concerns ovarian endometrioma and possibly severe/deep disease in relation to type I epithelial ovarian cancers, especially clear-cell and endometrioid carcinoma. Recent work also clarifies that EAOC is not a single entity: some tumors are genuinely endometriosis-correlated with transitional lesions, whereas others reflect incidental coexistence.

Molecular evidence supports biological plausibility, but current markers are not individual-level predictors for routine endometriosis care. Recent studies suggest that the microenvironment of endometriotic lesions plays an important role in this process. Iron-driven oxidative stress, hypoxia and inflammation may favor the survival of epithelial cells that have adapted to these conditions [10,11]. Clinical data further indicate that, once EAOC has developed, prognosis depends mainly on disease stage, the extent of cytoreduction and sensitivity to platinum-based chemotherapy, not on the presence of endometriosis itself [7,8]. The immediate clinical implication is risk stratification, integrating disease phenotype, histotype-specific evidence, hereditary cancer risk, menopausal status, imaging behavior, reproductive goals, and surgical trade-offs. In parallel, selected molecular alterations, particularly involving ARID1A and PIK3CA, may further inform risk assessment in a research setting, although their role in routine clinical stratification remains unestablished. The current evidence does not justify routine surveillance of all women with endometriosis. At the same time, the association with ovarian cancer should not be dismissed. A balanced approach is targeted awareness, careful evaluation of atypical ovarian lesions and research directed toward validated prediction models.

This review contributes to the literature by connecting three distinctions that are often considered separately: ovarian versus extraovarian endometriosis, endometriosis-correlated versus incidental cancer, and biological plausibility versus clinically validated prediction. Together, they explain why substantial relative risks and shared driver alterations do not justify treating every lesion as premalignant. Further progress requires longitudinal, phenotype-defined cohorts with standardized imaging, central pathology and matched molecular data. Such studies should estimate absolute risk over a specified time horizon and test whether adding molecular information improves decisions beyond age, menopausal status and lesion behavior. To date, evaluation of changing or atypical lesions remains more defensible than surveillance based on endometriosis status alone.

## Figures and Tables

**Table 1:** Key publications from 2024 to 2026 informing this clinical synthesis.

Publication	Design	Main Contribution	Clinical Interpretation
Leone Roberti Maggiore et al., 2026 [1]	State-of-the-art review	Integrated epidemiology, molecular data, imaging, biomarkers and clinical decision-making. Highlighted low absolute lifetime risk.	Core clinical synthesis; supports individualized care rather than universal surveillance.
Barnard et al., 2024 [4]	Population-based cohort	Endometriosis, especially endometrioma/DE, associated with higher ovarian cancer risk. The strongest risk for type I tumors.	Major phenotype-specific risk signal; not a screening mandate.
Piacenti et al., 2026/ENDOCANCER [2]	Systematic review/meta-analysis	OR 1.82, HR 3.03 and SIR 1.62 for ovarian cancer risk; absolute incidence at a level of 0.7–1.5	Quantitative synthesis; survival differences probably confounded by stage and histotype.
Bogani et al., 2026 [3]	Systematic/narrative multidisciplinary review	Relative risk generally modest; absolute lifetime risk usually <2–5%; routine surveillance/prophylactic surgery not supported.	Important clinical caution against overtreatment.
Moberg et al., 2026 [5]	Nationwide cohort	Highest SIRs for clear-cell and endometrioid ovarian cancer; combined endometriosis/adenomyosis exposure.	Confirms histotype specificity but requires caution due to detection bias.
Mezzapesa et al., 2025 [6]	Prospective observational study	Separated endometriosis-correlated from incidental ovarian cancer.	Prevents over-attribution of causality.
Beck et al., 2026 [7]	Real-world ESGO center cohort	Endometriosis status was not independently prognostic after adjustment.	Stage, tumor biology and cytoreduction remain dominant.
Sung et al., 2026 [8]	Retrospective single-center cohort (EAOC vs. HGSOC)	Better crude OS and PFS in EAOC, attenuated after stratification by stage and platinum sensitivity; no platinum-resistant EAOC patient survived beyond 5 years.	Prognosis depends mainly on stage, cytoreduction and chemosensitivity, not on endometriosis itself.
Ye et al., 2025 [9]	Molecular systematic review/meta-analysis	POLE-mutated, MMR-deficient and p53-abnormal patterns in EAOC.	Links EAOC to modern molecular classification.
Santhamma et al., 2026 [10]	Narrative review	Endometriosis as a clonal inflammatory disease; atypical endometriosis as the most plausible EAOC precursor; microenvironmental selection.	Conceptual framework for research on disease interception, not for screening.
Umezaki et al., 2026 [11]	Hypothesis and theory article	Coupled iron–hypoxia selection model with ferroptosis tolerance in the evolution of clear-cell carcinoma.	Testable mechanistic hypothesis with no new experimental data.
Yang et al., 2026 [12]	Single-cell and bulk transcriptomic reanalysis with TCGA-UCEC comparison	Compartment-specific inflammation–metabolism remodeling; myeloid signal not reproduced in bulk tissue.	Supports cell-resolved analyses and does not demonstrate a progression mechanism.
Dou and Xia, 2026 [13]	Integrative bioinformatics with in vitro validation	SLC12A8 upregulated in endometriosis and EAOC and associated with poorer EAOC prognosis.	Exploratory candidate biomarker.
Szubert et al., 2026 [14]	Systematic review	No reproducible miRNA profile suitable for clinical prediction.	Biomarkers remain exploratory.
Kordowitzki et al., 2026 [15]	Case–control study of blood-based aging biomarkers	Epigenetic age acceleration in endometriosis versus deceleration with telomere shortening in EAOC.	Small EAOC cohort; not a predictive marker.

## Data Availability

No new data were created or analyzed in this study. Data sharing is not applicable to this article.

## Abbreviations

The following abbreviations are used in this manuscript:

aHR	Adjusted hazard ratio
ART	Assisted reproductive technology
BRCA1/2	Breast cancer gene 1/2
CA-125	Cancer antigen 125
CI	Confidence interval
DE	Deep endometriosis
DNA	Deoxyribonucleic acid
EAOC	Endometriosis-associated ovarian cancer
ESGO	European Society of Gynaecological Oncology
FIGO	International Federation of Gynecology and Obstetrics
GPX4	Glutathione peroxidase 4
GSH	Glutathione
HE4	Human epididymis protein 4
HGSOC	High-grade serous ovarian carcinoma
HIF-1α	Hypoxia-inducible factor 1α
HNF-1β	Hepatocyte nuclear factor 1β
HR	Hazard ratio
JAMA	*Journal of the American Medical Association*
MAPK	Mitogen-activated protein kinase
miRNA	MicroRNA
MMR	Mismatch repair
MMRd	Mismatch repair deficiency
MRI	Magnetic resonance imaging
NRF2	Nuclear factor erythroid 2-related factor 2
OR	Odds ratio
OS	Overall survival
PI3K/AKT/mTOR	Phosphoinositide 3-kinase/AKT/mechanistic target of rapamycin pathway
PRISMA	Preferred Reporting Items for Systematic Reviews and Meta-Analyses
ROMA	Risk of Ovarian Malignancy Algorithm
ROS	Reactive oxygen species
SIR	Standardized incidence ratio
SLC7A11	Solute carrier family 7 member 11
SWI/SNF	SWItch/Sucrose Non-Fermentable chromatin remodeling complex
TCGA	The Cancer Genome Atlas
UCEC	Uterine corpus endometrial carcinoma
